# SEEKING AGE FRIENDLY UNIVERSITY DESIGNATION TAKES PATIENCE AND PERSISTENCE

**DOI:** 10.1093/geroni/igae098.1760

**Published:** 2024-12-31

**Authors:** Melissa Powers

**Affiliations:** University of Central Oklahoma, Edmond, Oklahoma, United States

## Abstract

The road to achieving Age Friendly University Designation at the University of Central Oklahoma was long and circuitous. It involved patience and persistence from the primary advocates. The initial steps involved gaining support on campus including from the Committee on Diversity, Faculty Senate, and Staff Senate. Both Senates unanimously approved resolutions in support of seeking the designation. In addition, the Senates added an affirmation that our campus community will be a place where the ideas and contributions of people of all ages are welcomed and valued. The next step on our roadmap was to gain approval from university leadership. It was at this level that our initial request stalled due to lack of interest. After a change in leadership, campus support was again evaluated and was determined to remain high. Due to the high level of campus support, the request was submitted directly to the President who approved seeking the designation without hesitation. The President specifically asked that our priority post designation be to conduct an age-friendly assessment of the campus to guide future priorities and programming. This assessment is currently underway. Our programming and priorities will be determined by the results of the age-friendly assessment.

